# Can increasing years of schooling reduce type 2 diabetes (T2D)?: Evidence from a Mendelian randomization of T2D and 10 of its risk factors

**DOI:** 10.1038/s41598-020-69114-8

**Published:** 2020-07-31

**Authors:** Charleen D. Adams, Brian B. Boutwell

**Affiliations:** 1 Havard T.H. Chan School of Public Health, 677 Huntington Ave, Boston, MA 02115 USA; 2 School of Applied Sciences, The University of Mississippi, P.O. Box 1848, University, MS 38677 USA; 3 John D. Bower School of Population Health, University of Mississippi Medical Center, Jackson, MS 39216 USA

**Keywords:** Metabolic disorders, Genetics, Risk factors

## Abstract

A focus in recent decades has involved examining the potential causal impact of educational attainment (schooling years) on a variety of disease and life-expectancy outcomes. Numerous studies have broadly revealed a link suggesting that as years of formal schooling increase so too does health and wellbeing; however, it is unclear whether the associations are causal. Here we use Mendelian randomization, an instrumental variables technique, with a two-sample design, to probe whether more years of schooling are causally linked to type 2 diabetes (T2D) and 10 of its attendant risk factors. The results revealed a protective effect of more schooling years against T2D (odds ratio = 0.39; 95% confidence interval: 0.26, 0.58; *P* = 3.89 × 10^–06^), which in turn might be partly mediated by more years of schooling being protective against the following: having a father with T2D, being overweight, having higher blood pressure and higher levels of circulating triglycerides, and having lower levels of HDL cholesterol. More schooling years had no effect on risk for gestational diabetes or polycystic ovarian syndrome and was associated with a decreased likelihood of moderate physical activity. These findings imply that strategies to retain adults in higher education may help reduce the risk for a major source of metabolic morbidity and mortality.

Tacit to most epidemiological research is a desire to infer whether an environmental exposure impacts some disease or health outcome in a causal fashion. A particular area of focus in recent decades, in particular, has involved examining the impact of educational attainment (years of schooling) on a variety of disease and life expectancy outcomes^1^. Numerous studies have broadly revealed a strong statistical association suggesting that as the years of formal schooling increase so too does health and wellbeing^2^. Indeed, educational attainment has been associated (in a protective sense) with diverse mental and physical health outcomes, including depression, cancer incidence, heart disease, and diabetes^1^.

Entangled in this line of inquiry—and much of social science research, in fact—is a concern about the degree of causal inference open to scholars^3^. With regard to the associations between educational attainment and health outcomes, Montez and Friedman caution: “Studies such as those highlighted above often implicitly assume that educational attainment has a causal influence on adult health; however, this assumption has long been challenged. If the assumption is incorrect then investing in education policies and schooling systems may waste government spending and not manifest in improved population health” (p. 1)^1^. To be sure, there is emergent evidence utilizing quasi-experimental and natural-experimental designs which suggest some causal effects may exist in some contexts for educational attainment on health outcomes^2^. Yet, there remains an overall dearth of evidence utilizing designs admitting of stronger causal inference capabilities.

More recently, scholars have begun utilizing data gleaned from large genomic consortia and publicly available genome wide association (GWA) studies to construct instrumental variables comprised of trait-relevant single-nucleotide polymorphisms (SNPs). When certain assumptions (discussed below) are satisfied in the data, it is possible to investigate whether some type of modifiable risk or protective factor causally impacts some outcome^4^. Known as Mendelian Randomization (MR), this variety of instrumental variable analysis has been increasingly applied to a range of medical and epidemiological outcomes^5^. In the current study, we apply MR modeling strategies to zoom in on whether educational attainment plays a causal role in the prevention of one of society’s most pressing public-health challenges: type 2 diabetes (T2D) and 10 of its risk factors.

## Results

### T2D

A strong protective effect against T2D is observed for more Education Years (odds ratio, OR, for T2D per SD increase in Education Years): IVW estimate 0.39; 95% confidence interval (CI) 0.26, 0.58; *P* = 3.89 × 10^–06^). The sensitivity estimators aligned in direction and magnitude of effects with the IVW’s estimate, and the MR-Egger intercept test indicated no evidence for directional pleiotropy. (Since this is also the case for all the tests—none showed evidence for directional pleiotropy with the MR-Egger intercept test, this statement will not be repeated for the remaining results. However, the *I*^2^ statistics, a measure of strength for the MR-Egger estimate^6–8^, indicated potential regression dilution for all tests. *I*^2^ statistics < 90% imply that the MR-Egger intercept test is susceptible to being false positive^6–8^. To account for this, simulation extraction (SIMEX) correction for dilution bias in the MR-Egger estimate and intercept^6,7^ were performed and are available in Supplementary Tables 12–22. SIMEX correction did not change the inferences for the tests of Education Years on T2D or any of the 10 risk factors).

### Sibling, mother, and father with diabetes

Small protective effects against having a sibling, mother, or father with diabetes are observed for more Education Years (ORs for a first-degree relative with diabetes per SD increase in Education Years): sibling IVW estimate 0.97; 95% CI 0.96, 0.98; *P* = 4.23 × 10^–11^); mother IVW estimate 0.97; 95% CI 0.96, 0.98; *P* = 6.66 × 10^–7^); father IVW estimate 0.98; 95% CI: 0.97, 0.99; *P* = 0.0008. The sensitivity estimators aligned in direction and magnitude of effects with the IVW’s estimate.

### Overweight status

A strong protective effect against being overweight is observed for more Education Years (OR for being overweight per SD increase in Education Years): IVW estimate 0.60; 95% CI 0.51, 0.72; *P* = 1.01 × 10^–08^). The sensitivity estimators mostly aligned in direction and magnitude of effects with the IVW’s estimate, with a slightly larger protective effect observed for the weighted mode estimator.

### Physical activity

A strong protective effect against performing the most amount of moderate physical activity is observed for more Education Years (OR for the highest level of moderate physical activity compared to all other amounts of moderate physical activity per SD increase in Education Years): IVW estimate 0.77; 95% CI 0.71, 0.84; *P* = 1.08 × 10^–08^). The sensitivity estimators varied in the magnitude of their effects, which might indicate unwanted pleiotropy.

### High blood pressure

A modest protective effect against having high blood pressure is observed for more Education Years (OR for high blood pressure per SD increase in Education Years): IVW estimate 0.94; 95% CI 0.92, 0.96; *P* = 2.49 × 10^–10^). The sensitivity estimators aligned in direction and magnitude of effects with the IVW’s estimate.

### Gestational diabetes and polycystic ovarian syndrome

There were null effects for the influence of Education Years on gestational diabetes and polycystic ovarian syndrome (OR for each per SD increase in Education Years): IVW estimate 1.00; 95% CI 1.00, 1.00; gestational diabetes, *P* = 0.1705; polycystic ovarian syndrome, *P* = 0.2844. The sensitivity estimators aligned in direction and magnitude of effects with the IVW’s estimate: all = 1.

### HDL levels

An increase in HDL levels were observed for more Education Years (beta estimate per SD increase in Education Years): IVW estimate 0.14; 95% CI 0.06, 0.22; *P* = 0.0009). The sensitivity estimators varied in the magnitude of effects, indicating the potential for some unwanted pleiotropy.

### Triglyceride levels

A decrease in triglyceride levels were observed for more Education Years (beta estimate per SD increase in Education Years): IVW estimate − 0.19; 95% CI − 0.27, − 0.11; *P* = 3.34 × 10^–06^). The sensitivity estimators aligned in direction and magnitude of effects with the IVW’s estimate (Table 1).

**Table 1:** Causal estimates for Education Years on T2D and 10 risk factors for T2D.

Test (No. SNPs)	R^2^	*F*	IVW			MR-Egger			MR-Egger intercept			Weighted median			Weighted mode		
			OR	95% CI	*P*	OR (*I*^2^)	95% CI	*P*	OR	95% CI	*P*	OR	95% CI	*P*	OR	95% CI	*P*
T2D (17)	0.003	13	0.39	0.26, 0.58	< 0.001*	0.38 (35%)	0.05, 3.09	0.381	1.00	0.96, 1.04	0.979	0.41	0.24, 0.69	< 0.001*	0.37	0.17, 0.80	0.022
Sibling with T2D (64)	0.008	38	0.97	0.96, 0.98	< 0.001*	0.95 (32%)	0.90, 1.00	0.044	1.00	0.99, 1.00	0.424	0.97	0.95, 0.98	< 0.001*	0.94	0.90, 0.98	0.005
Mother with T2D (62)	0.009	36	0.97	0.96, 0.98	< 0.001*	0.98 (27%)	0.93, 1.04	0.563	1.00	1.00, 1.00	0.734	0.98	0.97, 1.00	0.016	0.99	0.96, 1.02	0.567
Father with T2D (60)	0.008	37	0.98	0.97, 0.99	< 0.001*	1.00 (11%)	0.94, 1.06	0.984	1.00	1.00, 1.00	0.547	0.98	0.96, 0.99	0.006	0.98	0.94, 1.01	0.192
Over-weight (54)	0.007	20	0.60	0.51, 0.72	< 0.001*	0.61 (6%)	0.22, 1.74	0.364	1.00	0.98, 1.02	0.972	0.58	0.46, 0.73	< 0.001*	0.47	0.25, 0.91	0.029
Phys-ical activity (49)	0.006	37	0.77	0.71, 0.84	< 0.001*	0.87 (21%)	0.54, 1.40	0.568	1.00	0.99, 1.01	0.623	0.79	0.70, 0.91	< 0.001*	0.97	0.69, 1.36	0.852
High blood pressure (45)	0.006	36	0.94	0.92, 0.96	< 0.001*	0.94 (0%)	0.83, 1.07	0.369	1.00	1.00, 1.00	0.928	0.93	0.91, 0.96	< 0.001*	0.91	0.85, 0.97	0.005
Gest. diabetes (69)	0.009	38	1.00	1.00, 1.00	0.171	1.00 (35%)	1.00, 1.00	0.748	1.00	1.00, 1.00	0.547	1.00	1.00, 1.00	0.257	1.00	1.00, 1.00	0.332
POS (68)	0.009	38	1.00	1.00, 1.00	0.284	1.00 (36%)	1.00, 1.01	0.606	1.00	1.00, 1.00	0.460	1.00	1.00, 1.00	0.469	1.00	1.00, 1.00	0.831

Test (No. SNPs)	R^2^	*F*	IVW			MR-Egger			MR-Egger intercept			Weighted median			Weighted mode		
			*β*	95% CI	*P*	*β*	95% CI	*P*	*β*	95% CI	*P*	*β*	95% CI	*P*	*β*	95% CI	*P*
HDL levels (52)	0.007	12	0.14	0.06, 0.22	< 0.001*	0.35 (7%)	− 0.14, 0.84	0.171	− 0.004	− 0.012, 0.005	0.403	0.09	− 0.04, 0.21	0.163	0.004	− 0.27, 0.28	0.977
Trigly-ceridelevels (52)	0.007	23	− 0.19	− 0.27, − 0.11	< 0.001*	− 0.19 (6%)	− 0.67, 0.29	0.440	− 4.06E−06	− 0.008, 0.008	0.999	− 0.19	− 0.30, − 0.08	0.001	− 0.23	− 0.48, 0.03	0.087

T2D = type 2 diabetes; HDL = high-density lipoprotein cholesterol; Gest. diabetes = gestational diabetes; POS = polycystic ovarian syndrome; *P* = *P*-value; *F* = *F*-statistic; OR = odds ratio; CI = confidence interval. IVW = inverse-variance weighted test; IVW is the primary MR method. The MR-Egger, weighted median estimator, and weighted mode estimators are sensitivity tests for horizontal pleiotropy. If the magnitude and direction of their effects comport with those of the IVW estimate, this provides a screen against pleiotropy. The MR-Egger intercept is shaded grey because it is interpreted differently than the IVW estimate and the sensitivity estimators; the MR-Egger intercept provides a formal test for directional pleiotropy^9^. If the MR-Egger intercept is not different than 1 on the exponentiated scale or 0 when non-exponentiated (*P* > 0.05), this indicates a lack of evidence for bias due to pleiotropy in the IVW estimate.

*Indicates *P* < 0.005 (the Bonferroni threshold).

Table 2 contains the results of the mediation analysis of Education Years on T2D for seven of the risk factors.

### Sibling and mom with diabetes

No direct effect was observed for the influence of Education Years on T2D, when accounting for having either a sibling (OR 0.97; 95% CI 0.64, 1.46) or a mom with diabetes (OR 0.99; 95% CI 0.59, 1.64). The proportion of the total effect of Education Years on T2D potentially mediated by having a sibling or a mom with T2D is 96 and 98%, respectively. The confidence intervals cross 100 for both, which means the proportion mediated is not significant.

### Dad with diabetes

A direct effect Education Years on T2D remained accounting for having a dad with T2D (OR 0.52; 95% CI 0.30, 0.91). Having a dad with T2D potentially mediated 31% of the total effect (95% CI 19–43%).

### Overweight

A direct effect Education Years on T2D remained accounting for overweight status (OR 0.58; 95% CI 0.38, 0.88). Overweight status potentially mediated 42% of the total effect (95% CI 25–58%).

### HDL cholesterol

A direct effect Education Years on T2D remained accounting for higher HDL cholesterol (OR 0.50; 95% CI 0.36, 0.71). Overweight status potentially mediated 27% of the total effect (95% CI 16–37%).

### High blood pressure

A direct effect Education Years on T2D remained accounting for higher blood pressure (OR 0.43; 95% CI 0.28, 0.65). Overweight status potentially mediated 10% of the total effect (95% CI 6–14%).

### Triglycerides

A direct effect Education Years on T2D remained accounting for higher triglycerides (OR 0.44; 95% CI 0.30, 0.66). Overweight status potentially mediated 13% of the total effect (95% CI 8–19%).

**Table 2:** Mediation analysis of Education Years on T2D, exploring seven T2D risk factors as mediators.

Mediator	Effect	Odds ratio (OR)	Lower 95% CI	Upper 95% CI	*P* value	Proportion mediated (%) 95% CI
**Sibling with diabetes**						96 (58–134)
	Total	0.39	0.26	0.58	3.89E−06	
	Direct	0.97	0.64	1.46	8.67E−01	
**Mom with diabetes**						98 (59–137)
	Total	0.39	0.26	0.58	3.89E−06	
	Direct	0.98	0.59	1.64	9.45E−01	
**Dad with diabetes**						31 (19–43)
	Total	0.39	0.26	0.58	3.89E−06	
	Direct	0.52	0.30	0.91	2.11E−02	
**Being overweight**						42 (25–58)
	Total	0.39	0.26	0.58	3.89E−06	
	Direct	0.58	0.38	0.88	9.69E−03	
**HDL cholestero**l						27 (16–37)
	Total	0.39	0.26	0.58	3.89E−06	
	Direct	0.50	0.36	0.71	1.17E−04	
**High blood pressur**e						10 (6–14)
	Total	0.39	0.26	0.58	3.89E−06	
	Direct	0.43	0.28	0.65	7.21E−05	
**Triglycerides levels**						13 (8–19)
	Total	0.39	0.26	0.58	3.89E−06	
	Direct	0.44	0.30	0.66	5.82E−05	

## Discussion

We observed a protective effect of Education Years against T2D, which might be mediated in part by more years of schooling being protective against the following: having a first-degree relative with diabetes, being overweight, and having high blood pressure, higher levels of circulating triglycerides, and higher levels of HDL cholesterol. These findings comport with another causal study that examined education and diabetes with UK Biobank data. Davies et al. observed that leaving secondary school at an older age was causally protective against diabetes^10^. Their study differed from the present one in that ours examined education inclusive of college—Davies et al*.* focused on education up to college. Here, we document that the protective effect of education extends beyond schooling in adolescence. Years of schooling after high school decrease the chance of T2D^10^.

Our findings are also broadly in line with other recent MR studies examining education and some of these risk factors and the mediating effect of these on coronary heart disease (CHD). Moreover, Böckerman et al. found that education is a protective factor against obesity^11^. Carter et al*.* observed that blood pressure mediates the effect between education and coronary heart CHD^12^. And Tillman et al*.* found Education Years to be associated with favorable lipid profiles, where the impacts of Education Years on HDL cholesterol (beta estimate 0.15; 95% CI 0.07, 0.23) and triglycerides (beta estimate − 0.14; 95% CI − 0.22, − 0.06)^13^ tightly comport with the causal estimates we observed (HDL cholesterol beta estimate 0.14; 95% CI 0.06, 0.22; triglycerides beta estimate − 0.19; 95% CI − 0.27, − 0.11).

In the present study, more years of schooling had no effect on risk for gestational diabetes or polycystic ovarian syndrome and was associated with a decreased likelihood of moderate physical activity. Regarding the later, another recent MR study found little evidence that more education increased vigorous physical activity^14^. Thus, it seems unlikely that the protective effect of Education Years against T2D occurs through an influence on physical activity.

The protective effect against having a first-degree relative with diabetes is intriguing. The question becomes how best to interpret this finding. On one hand, several recent studies have documented that there is a bidirectional causal relationship between fluid intelligence and years of schooling^15,16^. While having higher fluid intelligence may causally impact more years of schooling, the magnitude of the effect for more years of schooling increasing fluid intelligence is comparatively larger: that is, the impact of Education Years on intelligence is more than two-fold greater than the impact of intelligence on Education Years^15,16^. Like educational attainment, which is sometimes treated as a proxy for cognitive ability, being brighter is protective against an array of negative health outcomes^17^. This means that it is possible that intelligence is confounding the present findings, especially those pertaining to a protective effect of more years of schooling against having a first-degree relative with diabetes. However, due to the durable influence of educational attainment on intelligence, it is also conceivable that those with more education might also positively influence their family members in ways that reduce risk for T2D.

In the formal mediation analysis, univariable MR demonstrated a strongly protective total effect of Education Years against T2D, and the individual multivariable MR analyses adjusting for genetic associations with having a father with diabetes, being overweight, having higher blood pressure, and higher HDL levels demonstrated protective but dampened direct effects of more Education Years. This suggests that interventions to increase Education Years would have larger net protective effect against T2D risk if they also had the consequences of reducing these established risk factors. No direct effect of Education Years was observed for having a sibling or a mother with T2D.

The influence of Education Years on reducing the chance of having first-degree relatives with T2D is small (the ORs are close to 1), so it is important to avoid over-interpretation of the effects. Nonetheless, assuming the findings replicate, there is an intriguing possibility which exists and should be further studied: “dynastic” effects might be somewhat responsible for these signals (see also^14^). For instance, there is a phenomenon that Kong et al*.* refer to as “genetic nurture”— the impact that non-transmitted parental alleles have on a child through impacts on parents and other relatives^18^. Kong et al*.* estimated the impacts of non-transmitted parental alleles on educational attainment of offspring and found that a polygenic score of the non-transmitted parental alleles was about 30% that of the polygenic score for transmitted alleles on educational attainment^18^. Moreover, they observed that the influence of non-transmitted alleles on various health-related traits of children was greater for mothers than fathers, supporting the intuitive notion that mothers have a greater nurturing impact than fathers. While they examined the uni-directional impact from parent to offspring, they speculate that a bidirectional relationship could exist and that siblings reciprocally influence each other. Though the magnitude of genetic nurturing from parent to offspring is likely to be much larger, is not impossible for the genome of a child to influence a first-degree relative through an influence on the environment^18^. Intuitively, this makes sense: as the child (and parent) ages, the parent naturally begins to increasingly depend on their offspring for healthcare assistance. “[T]he effects are likely to be bidirectional. For a parent–offspring pair, the magnitude of the effect in the direction of parent-to-offspring is likely to dominate the effect in the opposite direction. However, with siblings and twins, the effects would be reciprocal” (Kong^18^, p. 428).

Similarly, it is possible that some portion of the present findings are due to unaccounted for population structure. Though this is unlikely to be a major source of confounding, since the GWA studies used as data sources here addressed population stratification in their original designs, unaccounted for population stratification could cause a violation to the exclusion-restriction MR assumption and possibly induce associations between the Education Years instruments are confounders^19^. Future studies of Education Years and T2D and mediators of this relationship could be done in traditional family-based designs, which may be able to rectify some of this concern^20^.

A close companion of the concern for population stratification is a related problem that arises due to bias based on coincident geographic variation in genotype and health traits^21^. Haworth et al*.* demonstrated this form of latent structure with genetic data in the UK Biobank, similar to what was used here^21^. Triangulating (comparing) our results with those of other MR studies of similar traits provides a picture of consistency, however; while not eliminating the possibility, the triangulation of findings mitigates some of this concern.

Another limitation for the analyses of Education Years on a first-degree relative with diabetes is that it is possible that some cases of type 1 diabetes were included, since the UK Biobank questions that captured the measure for illnesses of relatives asked about “diabetes”—not specifically about T2D. However, the influence for this is expected to be minimal, since more than 90% of adults with diabetes have T2D^22^.

The primary limitation of the present study is one that all MR studies are liable to: unwanted horizontal pleiotropy. However, the most logical pleiotropic confounder—intelligence—is one that is influenced by Education Years. Moreover, most of the sensitivity screens for possible violations to the MR assumptions revealed little evidence for distortions due to pleiotropy. The exceptions are for HDL levels and physical activity, for which there was enough variability across the sensitivity estimators to view their results with more caution.

Our analytic set for T2D included some cases that were not of European decent^37^. While the Morris GWA study we used adjusted for population structure with genomic control, there is a remote possible that this may have impacted our findings.

A final potential limitation worth mentioning relates to the generalizability of the findings. Many of the T2D risk factors were assessed using UK Biobank participants, who do not represent the general UK population. On average UK Biobank participants are more health conscious and less likely to be socioeconomically deprived than the general population. That acknowledged, Fry et al*.* have reflected on the relevance of the differences: While estimates of disease incidence and prevalence obtained from UK Biobank data are unsuitable for generalizing, estimates of exposure-disease associations can be generalized to other populations^23^. Nonetheless, at a minimum, it is safest to restrict the generalizability of these findings to those of European ancestry and who are similar in socioeconomic advantage to those who volunteered for participation in the UK Biobank.

A strength of our study worth mentioning is that it leveraged the power of 11 large GWA studies to examine these complexly woven traits. Another strength is specific to the two-sample MR design. Genetic instruments that explain a small proportion of the variance in Education Years, perhaps leading to a weak instrument (indicated by small *F*-statistics), should bias the results towards the null and not towards a false-positive. This is important when considering, for instance, the findings for Education Years on HDL, where the *F*-statistic was 12^24,25^.

The public-health relevance of the bidirectional causal relationship between intelligence and Education Years cannot be overstated. If the present findings primarily reflect the benefits of higher cognitive ability—which they could—then whether Education Years influences cognitive ability informs interventional strategies. Because Education Years increases cognitive ability, public-health efforts to retain people in higher education may be warranted as part of a developing arsenal to help limit and even prevent the staggeringly deleterious effects of T2D. The message is the same, importantly, even if intelligence is not the driving force in the current study. In fact, the findings from another recent two-sample MR study, which investigated the impact of education and cognitive ability in a multivariable model in relation to CHD, provide evidence that intelligence is unlikely to be the important driver, at least in relation to CHD. Gill et al*.* found that more schooling years, independent of cognitive ability, are protective: OR 0.76; 95% CI 0.65–0.89^26^.

Whatever it is about the landscape of higher education, more years of schooling appears to help reduce the risk for major sources of metabolic morbidity and mortality. Our findings further contribute to the accumulating knowledge about this and could be used to stimulate policy discussions about increasing educational attainment in the general population. Increasing the number of years that people spend in the educational system may decrease their risk of developing T2D, and T2D that is attributable to lower levels of education may be reduced by intervening on some of the established T2D risk factors.

## Methods

### Conceptual approach

MR is an analytic, instrumental variables technique that capitalizes on Mendel’s Laws of Inheritance and genotype assignment at conception for causal inference^27–29^.

**Figure 1:**
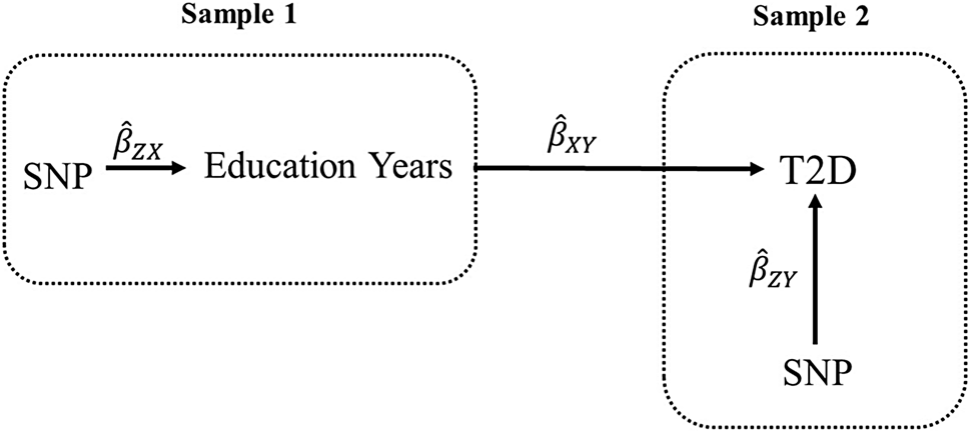
Two-sample MR testing the causal effect of Education Years on T2D. Estimates of the SNP-Education Years associations (*β*^ZX) are calculated in sample 1 (from a genome-wide association, GWA, study of Education Years). The association between these same SNPs and T2D is then estimated in sample 2 (*β*^ZY) (from a T2D GWA study). These estimates are combined into Wald ratios (*β*^XY = *β*^ZY/*β*^ZX). The *β*^XY estimates are meta-analyzed using the inverse-variance weighted analysis (*β*^IVW) method and various sensitivity analyses. The IVW method produces an overall causal estimate of Education Years on T2D.

MR uses genetic variants strongly associated with traits of interest as opposed to the observed traits themselves in models. By relying on the random assortment of alleles (Mendel’s Laws) and the temporal assignment of genotype at conception, MR avoids most sources of confounding and reverse causation that distort causal estimates in observational studies. In two-sample MR, summary statistics are pulled from two genome-wide association (GWA) studies. These summary statistics are the data sources for two-sample MR^4,9,30–33^ (Fig. 1).

### MR assumptions

MR has the following assumptions: (1) genetic instruments are strongly associated with the exposure; (2) genetic instruments are independent of confounders of the exposure and the outcome; and (3) genetic instruments are associated with the outcome only through the exposure^32,34^. For example, the following must be true in order for the present analysis to be valid: (1) genetic variants robustly associated with Education Years must be chosen as instruments to test the causal relationship between Education Years and T2D; (2) the genetic variants chosen to instrument Education Years must not be associated with confounders of the relationship between Education Years and T2D; and (3) the genetic variants chosen to instrument Education Years must only impact T2D through their impact on Education Years. When violated, assumption (3) describes horizontal pleiotropy, which can invalidate causal inference from vertical pleiotropy. Statistically based sensitivity estimators have been developed to evaluate potential violations to assumption (3) (for more on this, see the subsection, Sensitivity analyses).

### Design

This study explores the impact of Education Years on T2D and 10 risk factors for T2D. For the later, a list of established risk factors for T2D was obtained from the website for the American Diabetes Association (ADA) (https://www.diabetes.org/diabetes-risk)^35^:

- Being 45 or older
- Being Black, Hispanic/Latino, American Indian, Asian American, or Pacific Islander
- Having a parent with diabetes
- Having a sibling with diabetes
- Being overweight
- Being physically inactive
- Having high blood pressure
- Having low high-density lipoprotein (HDL) cholesterol
- Having high triglycerides
- Having had diabetes during pregnancy (gestational diabetes)
- Having been diagnosed with Polycystic Ovary Syndrome

Of these risk factors, all but “being 45 and older” and “being Black, Hispanic/Latino, American Indian, Asian American, or Pacific Islander” were suitable for investigation with two-sample MR.

### Exposure data source: Education Years

The instrument for Education Years was obtained from a GWA study of Education Years performed by Okbay et al. which included 293,723 participants of European ancestry and adjusted for 10 principal components, age, sex, and study-specific controls^36^. Education Years, inclusive of college, was measured for those who were at least 30 years of age. International Standard Classification of Education (ISCED) categories were used to impute a years-of-education equivalent (SNP coefficients per standard deviation, SD, units of years of schooling; an SD-unit of schooling = 3.6 years).

### Outcome data source: T2D

The outcome data for T2D was extracted from Morris et al*.* which performed a GWA study of T2D in 149,821 participants overwhelmingly of European decent, of which 34,840 had T2D. Their GWA adjusted for study-specific covariates and population structure^37^.

### Outcome data source: sibling with diabetes

The outcome data for having a sibling with diabetes was extracted from a GWA study performed by the Medical Research Council-Integrative Epidemiology Unit (MRC-IEU) staff, using PHESANT-derived^38^ UK Biobank data^39,40^ (UK Biobank data field 20,111). Briefly, the UK Biobank is an open-access cohort that enrolled about 500,000 participants, largely of European descent^41^. Genetic, health, and demographic data were collected on many of the participants and were made publicly available for researchers. The MRC-IEU staff ran numerous GWA studies with UK Biobank variables, adjusting for sex and genotyping chip, and used k-means cluster analysis for European ancestry (first four principal components, as provided by the UK Biobank)^42^. They made their results available through MR-Base, a public repository of summary statistics from GWA studies for use in MR analyses. The GWA study of having a sibling with diabetes contained 362,826 participants, of which 31,073 were classified as having a sibling with diabetes.

### Outcome data source: mother with diabetes

The outcome data for having a mother with diabetes was extracted from a GWA study performed by the MRC-IEU staff, which used PHESANT-derived UK Biobank data (UK Biobank data field 20110) and adjusted for sex and genotyping chip. They used k-means cluster analysis for European ancestry (first four principal components). The GWA study contained 423,892 participants, of which 40,091 were classified as having a mother with diabetes.

### Outcome data source: father with diabetes

The outcome data for having a father with diabetes comes from a GWA study performed by the MRC-IEU staff, which used PHESANT-derived UK Biobank data (UK Biobank data field 20107), adjusting for sex and genotyping chip, used k-means cluster analysis for European ancestry (first four principal components). The GWA study contained 400,687 participants, of which 38,850 were classified as having a father with diabetes.

### Outcome data source: overweight status

The outcome data for overweight status come from Berndt et al*.* which performed a GWA study of clinically defined overweight status in 158,855 participants of European ancestry, of which 93,015 were classified as overweight^43^. Overweight case status was defined as BMI ≥ 25 kg/m^2^.

### Outcome data source: physical activity

The outcome data for physical activity come from a GWA study by the MRC-IEU staff, which used PHESANT-derived UK Biobank data for moderate physical activity, defined as the number of days of moderate physical activity per week performed for more than 10 min at a time. The GWA study included 440,266 participants and was adjusted for sex and genotyping chip, with k-means cluster analysis for European ancestry (first four principal components).

### Outcome data source: high blood pressure

A GWA study of high blood pressure (a binary measure) was performed by the MRC-IEU staff using PHESANT-derived variables^38^ constructed from the UK Biobank data^39,40^ (data field 6150: “Vascular/heart problems diagnosed by doctor: high blood pressure”), which adjusted for sex and genotyping chip and used k-means cluster analysis for European ancestry (first four principal components). There were 461,880 participants, of which 124,227 had high blood pressure as determined by a physician.

### Outcome data source: gestational diabetes

The GWA study of gestational diabetes (a binary measure) was performed by MRC-IEU staff using PHESANT-derived variables^38^ constructed from UK Biobank data^39,40^ (data field 4041), adjusting for sex and genotyping chip and with k-means cluster analysis for European ancestry (first four principal components). Participants were asked if they only had diabetes during pregnancy. There were 462,933 participants, 240 of which self-reported having had gestational diabetes.

### Outcome data source: polycystic ovarian syndrome

The outcome data for polycystic ovarian syndrome (a binary measure) was performed by MRC-IEU staff using PHESANT-derived variables^38^ constructed from the UK Biobank data^39,40^ (data field 20002), adjusting for sex and genotyping chip, with k-means cluster analysis for European ancestry (first four principal components). There were 462,933 participants, of which 571 self-reported having polycystic ovarian syndrome.

### Outcome data source: HDL levels

The outcome data for circulating HDL levels (a continuous measure) come from Willer et al*.* which performed an age- and sex-adjusted GWA study of circulating HDL levels in up to 187,167 individuals, largely of European ancestry^44^.

### Outcome data source: triglyceride levels

The outcome data for triglyceride levels (a continuous measure) come from Willer et al*.* which performed an age- and sex-adjusted GWA study of circulating triglyceride levels in up to 177,861 individuals, largely of European ancestry. They adjusted for population structure^44^.

To ease interpretability, all MR results for the effects of Education Years on T2D and T2D risk factors were exponentiated from log odds to odds ratios, except for outcomes of continuous variables (i.e., HDL and triglyceride levels), which are presented as beta estimates (Table 1).

The summary statistics used for the MR analyses are available in Supplementary Tables 1–11.

### Instrument construction

As introduced in Fig. 1, independent (those not in linkage disequilibrium, LD; R^2^ < 0.001) SNPs associated at genome-wide significance (*P* < 5 × 10^–8^) with Education Years were extracted from the Okbay et al.^36^*.* GWA study. The summary statistics for the Education Years-associated SNPs were then extracted from each of the outcome GWA studies. SNP-Education Years and SNP-outcome associations were harmonized and combined with the IVW method using first-order weights (Fig. 1).

### Sensitivity analyses

A weakness of the IVW estimator is that its estimate can be biased if the meta-analyzed SNPs are directionally pleiotropic^45^. This can cause a violation to MR assumption (iii) and invalidate the findings. To address this, MR-Egger regression, weighted median, and weighted mode MR methods can be run as complements to the IVW. The directions and magnitudes of their effect estimates can be compared to those of the IVW. Doing so is a type triangulation: comparing approaches that have different assumptions to weigh evidence^46^. The reason for this is that the various MR sensitivity estimators make different assumptions about possible underlying pleiotropy. Due to their different assumptions, it is unlikely that the IVW and sensitivity estimators would be homogeneous in the directions and magnitudes of their effect estimates if there were substantial violations to MR assumption (iii). Therefore, triangulating their directions and magnitudes of effects provides a screen against pleiotropy. (Nuanced descriptions of how the various MR estimators deal with pleiotropy are described elsewhere^45,47,48^). MR-Egger regression, weighted median, and weighted mode MR sensitivity methods were run for all analyses.

A formal test for directional pleiotropy was also done with the MR-Egger intecept. If the MR-Egger intercept is not different than 1 on the exponentiated scale or 0 when non-exponentiated (*P* > 0.05), this indicates a lack of evidence for bias due to pleiotropy in the IVW estimate.

A SIMEX correction procedure that adjusts the MR-Egger estimate for potential regression dilution to the null^6^ was performed for all tests. When the *I*^2^ statistic is < 90%, correction procedures are recommended. SIMEX correction was applied for all tests.

In addition, potential outlier SNPs were removed using RadialMR regression^49^ for the MR tests of Education Years on T2D risk factors. (The differing number of SNPs for the Education Years instruments is due to this and that the various outcome GWA studies not having a uniform set SNPs in their association studies). All instrumental variables included in this analysis have Cochrane’s *Q*-statistic *P* values indicating no evidence for heterogeneity between SNPs^50^. Heterogeneity in the effect estimates for SNPs can indicate pleiotropy. Thus, ensuring a lack of heterogeneity between SNPs is an additional method to boost the chance that MR assumption (iii) is not violated. Heterogeneity statistics, forest and scatter plots, and the results of the SIMEX correction are provided in Supplementary Tables 12–22.

The IVW and sensitivity estimations were performed in R version 3.5.2 with the “TwoSampleMR” package^30,51^. Overall, 11 tests were performed. The Bonferroni correction was used to penalize for multiple testing: *P* = 0.05/11 (0.005).

### Power

**Figure 2:**
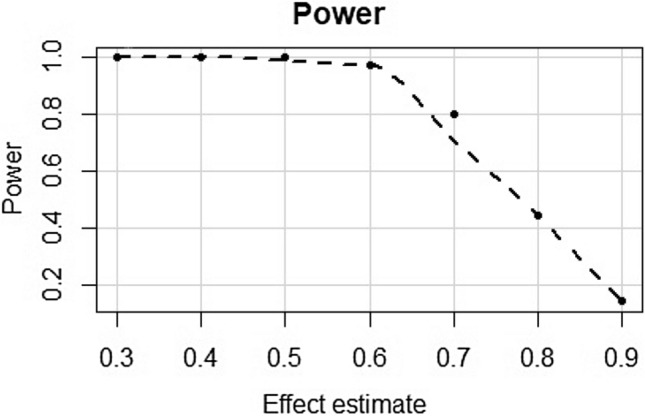
Power calculations for a range of plausible effects estimates for the MR test of Education Years on T2D.

The study was powered for the test of Education Years on T2D, using mRnd MR power calculator (available at https://cnsgenomics.com/shiny/mRnd/)^52^. There was ≥ 80% power to detect odds ratios in the range of 0.3–0.7 (Fig. 2). In addition to the overall power to detect an association, MR studies also rely on *F*-statistics. *F*-statistics provide an indication of instrument strength^53^. *F*-statistics < 10 are conventionally considered to be weak^24^. *F*-statistics for each test are available in Table 1.

### Formal mediation

Based on the results of the univariate models of Education Years on T2D and the 10 risk factors, a formal appraisal of mediation was performed for seven of the 10 risk factors: first-degree relative with diabetes, being overweight, and having high blood pressure, higher HDL cholesterol, and more triglycerides. Although not the focus of this investigation, univariate models of the effects of these established risk factors on T2D were run to identify potential outliers to remove in the multivariate analyses (only SNPs not observed to be outliers in the univariable analyses were kept for the multivariable analyses), using the same data sources and methods as above. Supplementary Tables 23–29 contain the summary statistics used for these analyses, and Supplementary Tables 30–36 contain the results for the sensitivity analyses, heterogeneity statistics, forest and scatter plots, the results of the SIMEX corrections, and the results of the multivariate analyses.

With traditional regression-based mediation analyses, three parameters are usually estimated (i) the total effect (the effect of the exposure on the outcome) (ii) the direct effect (the effect of the exposure on the outcome that is not through the mediator) and (iii) the indirect effect (the effect of the exposure on the outcome that occurs through the mediator)^54^.

With MR, mediation analysis can be done by generating the total effect with univariate MR and the direct effect with multivariate MR. The indirect effect is calculated by subtracting the direct effect from the total effect. The proportion of the total effect that is mediated is calculated by dividing the indirect effect by the total effect^54^.

Typically, with individual-level MR data, bootstrapping can be done to estimate confidence intervals for the indirect effect and the proportion mediated^54^. However, since the analysis at hand was performed using summary data, estimates of variability for the proportion mediated were calculated with the delta method. The calculations for the indirect effects and the proportion mediated were performed on the log odds scale (Supplementary Table 37), and the results were then exponentiated to odds ratios. The multivariable MR analyses were performed using the “mv_multitiple” function for generating IVW estimates within the “TwoSampleMR” package, after clumping for LD and harmonizing^19^.

Final notes about the Methods used here. A previous MR study^55^ also looked at Education Years on T2D with the Okbay GWA source for Education Years and the Morris GWA source for T2D^55^. To clarify, our analysis differed from theirs, as we used the “Metabochip” set from Morris, which included more samples. Moreover, the Hagenaars analysis was bidirectional, which has assumptions not relevant to the analysis at hand. Davies et al.^14^ also examined Education Years on T2D, though they used samples in the UK Biobank (not the Morris GWA study) for their T2D data source^14^.

No human subjects or tissues were used for the analyses presented in this study, nor were any experiments conducted. Informed consent for the GWA sources accessed here was previously reported and obtained by the cohorts that generated the primary data^36,37,39,40,43,44^, as is standard. No institutional approvals were needed to perform the analyses reported here, since the data sources are public and secondary in nature (i.e., they are summary-level statistics; no individual-level data was acquired or generated). This study was conducted in accordance with best practices for MR and follows the “Strengthening the Reporting of Observational Studies in Epidemiology” (STROBE) reporting guidelines, where pertinent for MR.

## Supplementary information

Supplementary file1 (XLSX 1416 kb)

## Data Availability

All data sources are publicly available and are accessible within MR-Base: https://www.mrbase.org/^30^.
